# Visualization of Glutamine Transporter Activities in Living Cells Using Genetically Encoded Glutamine Sensors

**DOI:** 10.1371/journal.pone.0038591

**Published:** 2012-06-14

**Authors:** Katrin Gruenwald, John Todd Holland, Verlyn Stromberg, Altaf Ahmad, Daisy Watcharakichkorn, Sakiko Okumoto

**Affiliations:** 1 Department of Plant Pathology, Physiology and Weed Science, Virginia Tech, Blacksburg, Virginia, United States of America; 2 Department of Botany, Faculty of Science, Hamdard University, New Delhi, India; University of Iowa, United States of America

## Abstract

Glutamine plays a central role in the metabolism of critical biological molecules such as amino acids, proteins, neurotransmitters, and glutathione. Since glutamine metabolism is regulated through multiple enzymes and transporters, the cellular glutamine concentration is expected to be temporally dynamic. Moreover, differentiation in glutamine metabolism between cell types in the same tissue (e.g. neuronal and glial cells) is often crucial for the proper function of the tissue as a whole, yet assessing cell-type specific activities of transporters and enzymes in such heterogenic tissue by physical fractionation is extremely challenging. Therefore, a method of reporting glutamine dynamics at the cellular level is highly desirable. Genetically encoded sensors can be targeted to a specific cell type, hence addressing this knowledge gap. Here we report the development of Föster Resonance Energy Transfer (FRET) glutamine sensors based on improved cyan and yellow fluorescent proteins, monomeric Teal Fluorescent Protein (mTFP)1 and venus. These sensors were found to be specific to glutamine, and stable to pH-changes within a physiological range. Using cos7 cells expressing the human glutamine transporter ASCT2 as a model, we demonstrate that the properties of the glutamine transporter can easily be analyzed with these sensors. The range of glutamine concentration change in a given cell can also be estimated using sensors with different affinities. Moreover, the mTFP1-venus FRET pair can be duplexed with another FRET pair, mAmetrine and tdTomato, opening up the possibility for real-time imaging of another molecule. These novel glutamine sensors will be useful tools to analyze specificities of glutamine metabolism at the single-cell level.

## Introduction

Glutamine is essential as the precursor for other amino acids such as glutamate, histidine, proline and arginine, as well as many other important biological molecules such as proteins, nucleic acids [1], amino sugars [2] and glutathione [3], [4]. It is also a preferred fuel for rapidly dividing cells such as enterocytes, fibroblasts and lymphocytes [5], [6], and it serves as an important precursor for neurotransmitters glutamate and GABA [7], [8], [9], [10].

Because glutamine occupies a central position in primary and secondary metabolism, glutamine availability has a large impact on anabolism of downstream molecules. For example, recent studies using an epileptic model brain suggested that the availability of glutamine influences the amount of synaptically released glutamate [11], [12]. Moreover, in addition to its roles as an anabolic precursor, evidence suggests that glutamine has regulatory roles over many cellular functions such as cell swelling-induced signaling [13] and apoptosis [14]. Recent studies suggest that glutamine induces a drastic change in gene expression (∼1% of analyzed genes) in pancreatic β-cell lines [15], as well as when administered as a dietary supplement. Therefore, the cellular glutamine level has a large impact on cell physiology, through the regulation of both glutamine-derived molecules, and glutamine-controlled cellular functions.

The control of cellular glutamine levels is a very complex process. The activity of glutamine synthetase, the enzyme that synthesizes glutamine from glutamate and ammonium, is regulated by a wide range of mechanisms including allosteric regulation by substrates [16], assembly of subunits [17] and transcriptional regulation by glucocorticoids and β-catenin [18], [19], [20]. Glutaminase and glutamine transporters such as system N and ASCT transporters are subject to both transcriptional and post-transcriptional regulation [21], [22], [23]. In addition to the environmental regulation exerted by the above mechanisms, the regulation of glutamine metabolism is highly cell-type specific. In the mammalian liver, two separate cell types are involved in a sequential glutamine degradation and synthesis; the cells in the periportal region generate ammonium from glutamine to provide ammonia necessary for the production of urea, whereas in the perivenous hepatocyte, glutamine is synthesized by glutamine synthetase to scavenge excess ammonia that escaped the urea cycle [24], [25], [26]. Cellular specialization of glutamine metabolism is also found in neuronal tissues of animals. Glutamate released from the neuronal cells is taken up by the surrounding glial cells and converted into glutamine, which is not a transmitter molecule. The synthesized glutamine is then shuttled back to the neuron where it is degraded into glutamate to replenish the neurotransmitter pool. This so-called glutamine-glutamate shuttle is considered to be important in sustaining neuronal activities [7], [8].

Because of the multiple levels of regulation and the heterologous functions of different cell types, it is not surprising that the concentration of glutamine varies greatly between cell types. For example, glutamine is found in high concenrations in oligodendrocytes and astrocytes (22 mM), but at much lower concentrations in glutamatergic terminals (4–11 mM) [27]. Likewise, in hepatocytes glutamine is reported to be 20 mM, but the stimulation of glutaminase or glutamine transaminase leads to a decrease of glutamine concentration by 80% and 30%, respectively [28]. In cancerous cells with activated glutamine catabolism, the glutamine concentration was found to be much lower than in cells with a lower rate of glutamine catabolism [29].

Genetically encoded sensors targeted to a single cell type or to a subcellular compartment would provide an alternative approach to analyze the dynamic regulation of metabolites in a single cell. A set of FRET sensors for glutamine has previously been reported, and was successfully used to monitor glutamine concentrations in plant cells [30]. However, these sensors were limited in the range of glutamine concentration that can be detected due to relatively low affinity (6.8 and 18.8 mM). Also, enhanced cyan- and yellow- fluorescent protein (ECFP and EYFP) were used as the FRET pair, which is difficult to multiplex with other FRET pairs. Here we report an array of improved FRET-based glutamine sensors based on the *E.coli* glutamine binding protein, glnH. The sensors consist of a recently reported FRET pair, monomeric Teal Fluorescent Protein (mTFP)1 and venus. Both mTFP1 and venus have improved quantum efficiency and pH stability compared to the more commonly used FRET donor, ECFP and EYFP [31], [32]. Moreover, the mTFP1-venus FRET pair can be duplexed with another pair, mAmetrine-tdTomato [33]. We demonstrate that the glutamine uptake and efflux can be monitored using these glutamine sensors, making it an attractive tool to analyze the properties of transporters expressed in a given cell. The properties of transporters such as substrate specificity and dependency on sodium gradient could easily be monitored. In addition, using sensors with different affinities, we show that a wide range of cytosolic glutamine concentrations can be monitored.

## Results and Discussion

### FRET Glutamine Sensor Using mTFP1-Venus FRET Pair

Previously developed glutamine sensors using the CFP/YFP FRET pair are not compatible with other protein-based FRET pairs because of substantial excitation/emission spectra overlap [34]. In addition, the excitation maxima of CFP (428 nm) is not ideal for imaging using confocal microscopy. Recently, Ai *et al.* reported an improved cyan fluorescent protein (FP) from coral, mTFP1, which has higher quantum efficiency and improved pH stability [31]. Moreover, two protein-based FRET pairs, mTFP1/Citrine (enhanced Yellow FP) and mAmetrine/tdTomato are spectrally orthogonal and therefore can be used for dual-FRET measurement [35]. We examined whether glnH (accession: NP_415332), the high affinity glutamine binding protein from *E.coli*, could be converted into a FRET sensor using mTFP1 and an improved yellow FP, venus [32].

**Figure 1:**
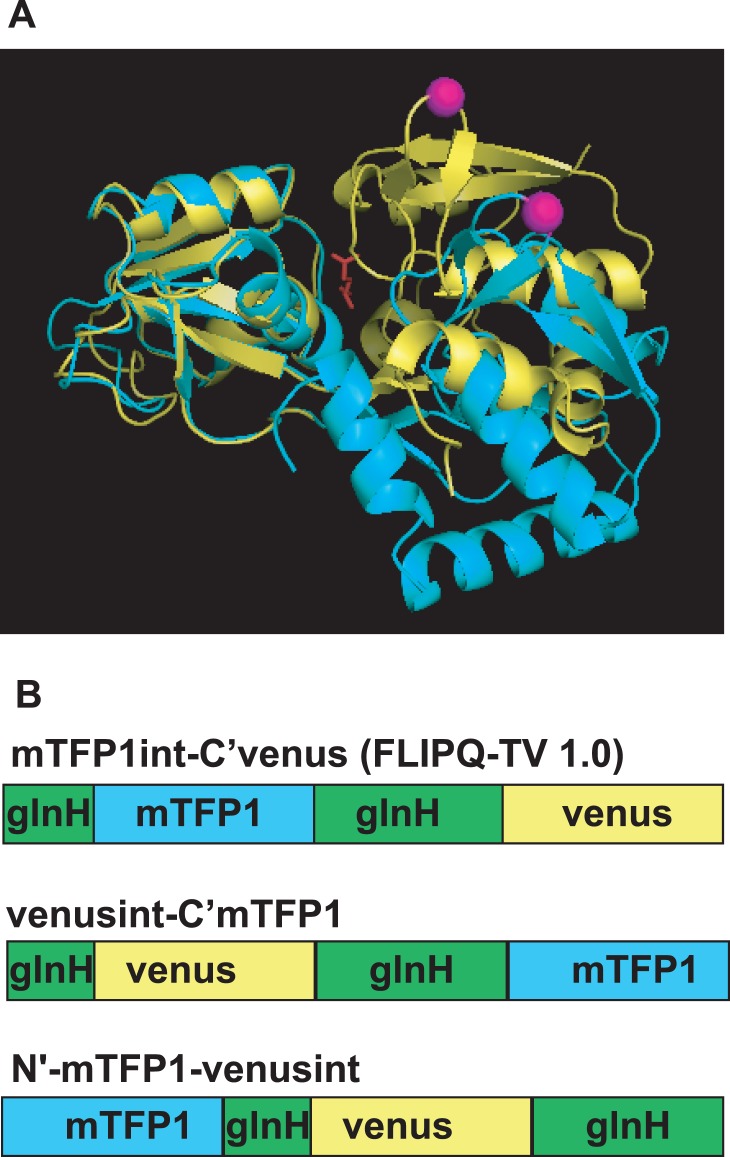
Configuration of a FRET glutamine sensor. (A) Open (cyan) [36] and closed (yellow, glutamine in the binding pocket is indicated in red) [37] conformation of glnH, glutamine binding protein from *E.coli*. The position of the internal hairpin permissive to an insertion of FP is marked in magenta. (B) Schematic representations of chimeric fusions between mTFP1, glnH and venus sequences.

The sites of attachment for the donor and acceptor molecules influence FRET efficiency, because they influence both the distance between fluorophores and dipole-dipole orientation. The crystal structure of glnH in both the open and closed form has been published previously [36], [37]. One of the lobes of glnH contains a large hairpin-like structure close to its N terminus, which allows insertion of the FPs (Fig. 1A, the permissive position is indicated in magenta). It has previously been demonstrated that insertion of ECFP in the corresponding location in the glutamate binding protein ybeJ, which is structurally related to glnH, does not interfere with the binding of glutamate to the chimera protein [38]. Therefore, we systematically tested combinations of these three possible insertion sites for the donor and acceptor proteins (Fig. 1B). For venus, it is known that terminal regions (an N-terminal helix and a C-terminal coil) are not required for the fluorescence [38]. Therefore, in addition to full-length venus, a series of clones that had part of the N- and C- terminal amino acids removed were used in order to find the optimal linker length. Among the constructs examined, we found one functional mTFP1/venus based glutamine sensor, named FLIPQ-TV(m**T**FP/**V**enus) 1.0 (Fig. 1B). In this configuration, mTFP1 and venus are located in the same lobe, hence the conformational change in the glnH domain is unlikely to induce a significant change in the distance between the two FPs. However, the binding of glutamine causes a shift of the second lobe, opening up a larger space in the vicinity of C-terminus where the venus molecule is fused (Fig.S1). Such a change is likely to increase the accessible space that the venus protein can occupy due to the decrease in sterical constraint, hence affecting the FRET efficiency between the two FPs. In fact, a number of type II periplasmic binding proteins, in which N- and C- termini are located in the same lobe, can be converted into a functional FRET sensors when the two FPs are fused on the N- and C- termini [38], [39], [40].

**Figure 2:**
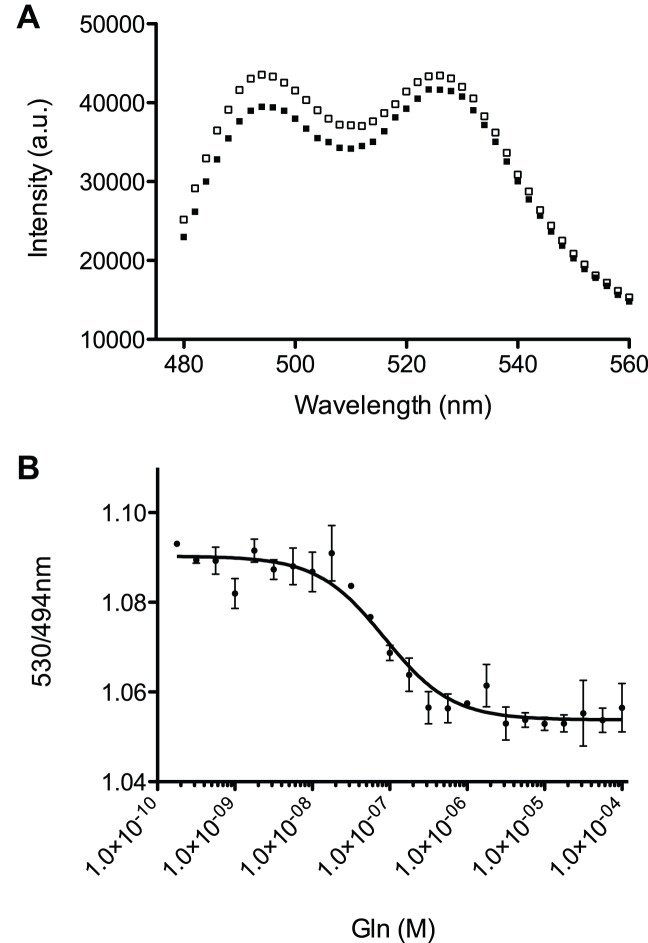
Responses of FLIPQ-TV1.0 sensor to glutamine. (A) Emission spectra of the FLIPQ-TV1.0 sensor in the absence (black squares) or in the presence of 1 mM glutamine (open squares). A.u. : arbitrary unit. (B) Concentration-dependent change of venus/mTFP1 peak intensity ratio. The best fit to a single binding isotherm (R = R_max_ - (R_max_-R_min_) x [L]/([*Kd*]+[L]), where R is ratio, R_max_ and R_min_ are the maximum and minimum ratios respectively, and [L] is ligand concentration) is indicated.

The emission from the donor and acceptor changed reciprocally when glutamine was added, indicating that the binding of the substrate is transduced to the change in FRET efficiency (Fig. 2A). The change in FRET efficiency was concentration dependent. The approximate *Kd* of this sensor (8.56±1.43×10^−8^ M) was consistent with the previously published affinities of glnH (1×10^−8^ to 3×10^−7^ M) (Table 1 and Fig. 2B) [40], [41], [42]. These results indicate that glnH can be converted into a FRET sensor using the mTFP1/venus FRET pair.

**Table 1:** Affinities of FLIPQ-TV3.0 point mutants.

Mutations in glnH	*Kd*	R_apo_	R_sat_	ΔR/R_0_
WT	85 nM	1.09	1.05	0.03
R75K	1.5 µM	1.23	0.90	0.26
R75M	50 µM	1.05	0.95	0.10
D157N	130 µM	1.34	0.99	0.26
R75MY86A	1.6 mM	1.29	1.13	0.12
R75MW220A	7.6 mM	1.14	1.01	0.11

**Figure 3:**
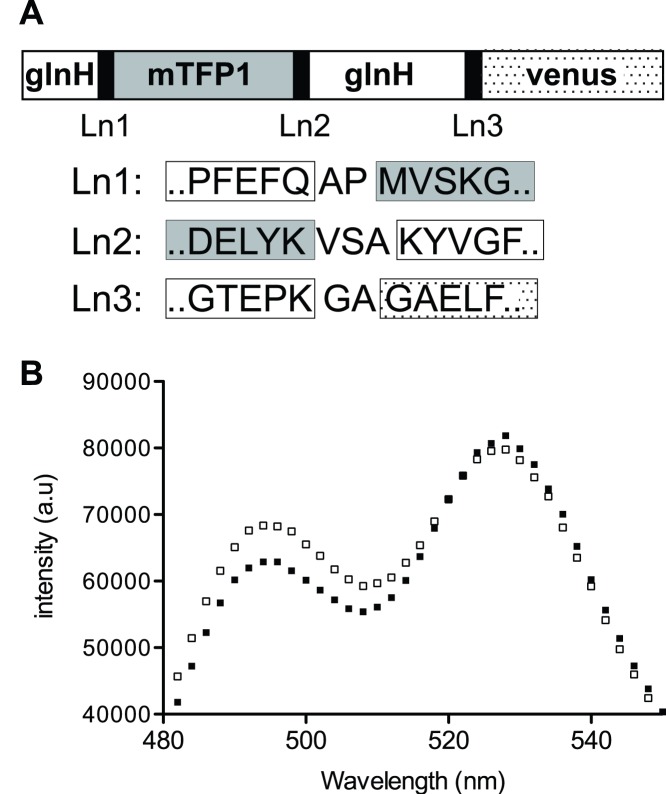
Improvement of the FLIPQ-TV sensor through semi-high throughput screening. (A) Schematic representation of FLIPQ-TV 3.0_R75K sensor, in which linker sequences (indicated as Ln1–3) were sequentially altered through random mutagenesis. The linker sequence of the resulting clone (FLIPQ-TV 3.0_R75K) is indicated. (b) Emission spectra of the FLIPQ-TV3.0_R75K sensor in the absence (black squares) or in the presence of 1 mM glutamine (open squares).

**Figure 4:**
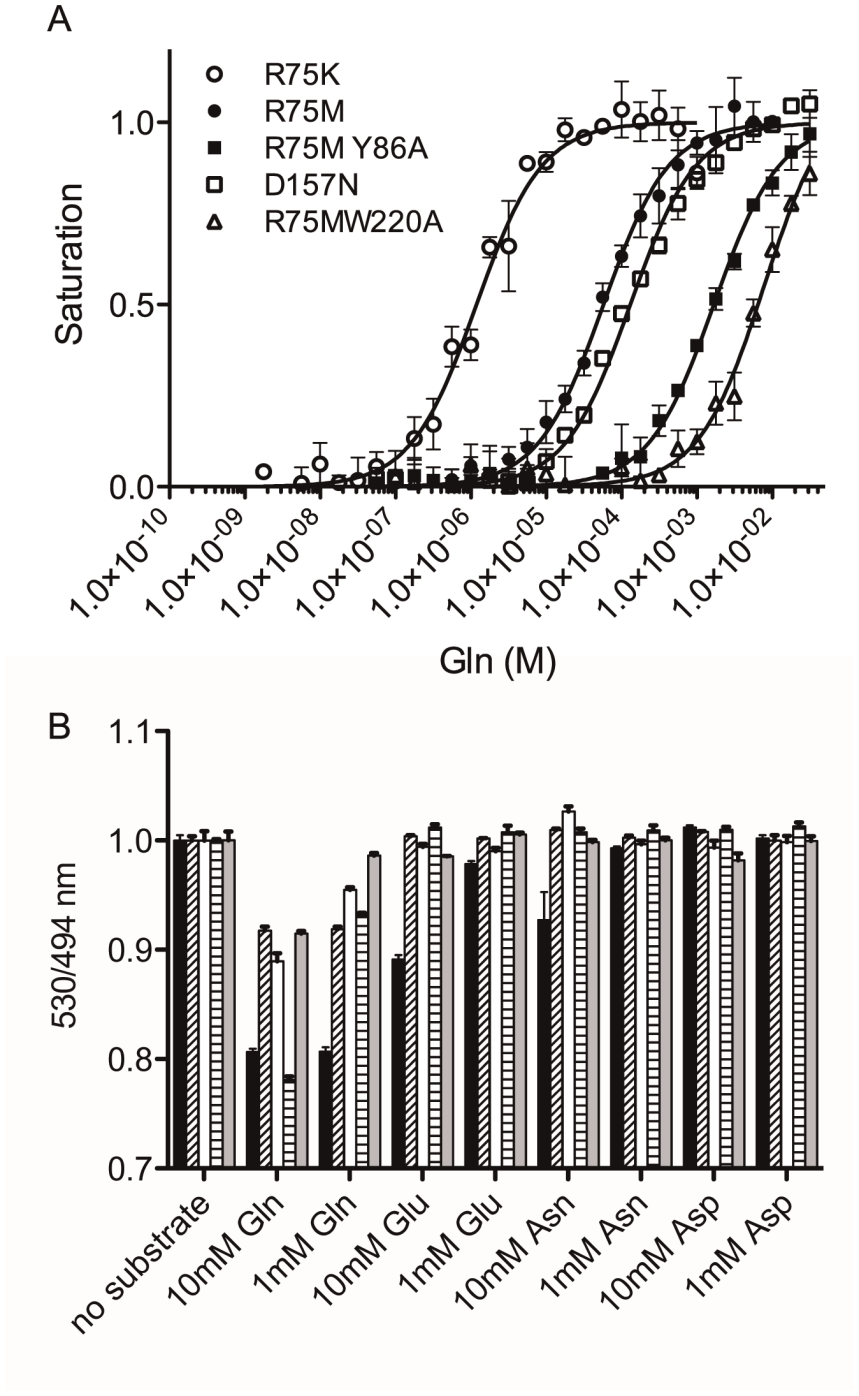
Affinities and substrate specificities of FLIPQ-TV3.0 sensors. (A) Saturation curves of FLIPQ-TV3.0 sensors with altered affinities. (B) Substrate specificities of FLIPQ-TV3.0_1.5 μ (black), 50 μ (hatched), 100 μ (white), 2 m (horizontal stripes), and 8 m (gray) sensors to Gln, Glu, Asn and Asp.

The FLIPQ-TV1.0 had very low FRET efficiency change upon binding of glutamine (Fig. 2B, ΔR/R_0_ = 0.033). To further improve the FRET efficiency change of FLIPQ-TV sensors, linker sequences between the binding protein and fluorophores were modified using a semi-high throughput approach. Linker sequences at the N- and C- termini of mTFP1, and N- terminus of venus were altered sequentially through random mutagenesis (Fig. 3A and Fig.S2) to select for clones with an improved FRET efficiency change. In order to avoid the potential saturation of the sensors due to contamination by glutamine from bacterial lysate, a version of the sensor that had ∼20 times lower affinity compared to the sensor based on wild-type glnH (FLIPQ-TV_R75K, Table 1 and Fig. 4A) was used as the starting clone for the optimization.

In each round, >700 clones were screened for an improved FRET efficiency change (details of mutagenesis and screening procedures are described in Fig. S2.). The sequential improvement yielded FLIPQ-TV3.0_R75K, which had >4 fold improvement compared to FLIPQ-TV1.0_R75K(Fig. 3B and Fig.S2, ΔR/R_0_ = 0.26).

### Affinity and Substrate Specificities of FLIPQ-TV3.0 Sensors

Reported physiological glutamine concentrations in the cytosol vary significantly (22 mM oligodendrocytes and astrocytes, 4–11 mM in glutametergic terminals, [27]. 20 mM in hepatocytes [28], 2.7–2.8 mM in hippocampus [43]). In order to create sensors that have a dynamic range at all physiological concentrations of glutamine, targeted site-directed mutagenesis was performed. Among the residues tested, R75, which forms a hydrogen bond with the α-carboxyl group of glutamine [37], resulted in significant changes in the affinity when mutated into K and M. Also, D157 which forms a hydrogen bond with α-amino group [37], when mutated into N, resulted in a variant with a lower affinity (Fig. S3A). In addition to the mutations in the binding pocket, mutations in the residues located either at the perimeter of the interdomain cleft (peristeric) or in the domain that undergoes a significant local conformation change upon substrate binding (allosteric) can cause changes in affinity [41]. Among such residues identified by de Lorimier *et al.,* Y86 (allosteric) and W220 (peristeric) altered the affinity when mutated into A (Fig. S3B). The resulting clones, FLIPQ-TV3.0_R75K, R75M, D157N, R75MY86A, and R75MW220A had *Kd* of 1.5×10^−6^ M, 5.3×10^−5^ M, 1.3×10^−4^ M, and 1.6×10^−3^, and 7.6×10^−3^ M, respectively (Fig. 4A, Table 1). These sensors were named FLIPQ-TV3.0_1.5 μ, 50 μ, 100 μ, 2 m, and 8 m (the numbers in names were rounded off for the simplicity). The complete list of residues that were tested for altered affinity can be found in Table S1.

The affinity of these sensors to similar amino acids has also been tested. FLIPQ-TV3.0_1.5 μ bound to glutamate and asparagine at concentrations higher than 1 mM and 10 mM, respectively (Fig. 4B). The *Kd*s to glutamate and asparagine could not be reliably measured due to its low affinity, estimated to be >10 mM in both cases. FLIPQ-TV3.0_50 μ, 100 μ, 2 m and 8 m did not bind to glutamate, asparagine or aspartate at 10 mM concentrations (Fig. 4B). From these results, we concluded that FLIPQ-TV3.0 sensors are highly specific to glutamine.

### pH Stability of FLIPQ-TV3.0 Sensors

**Figure 5:**
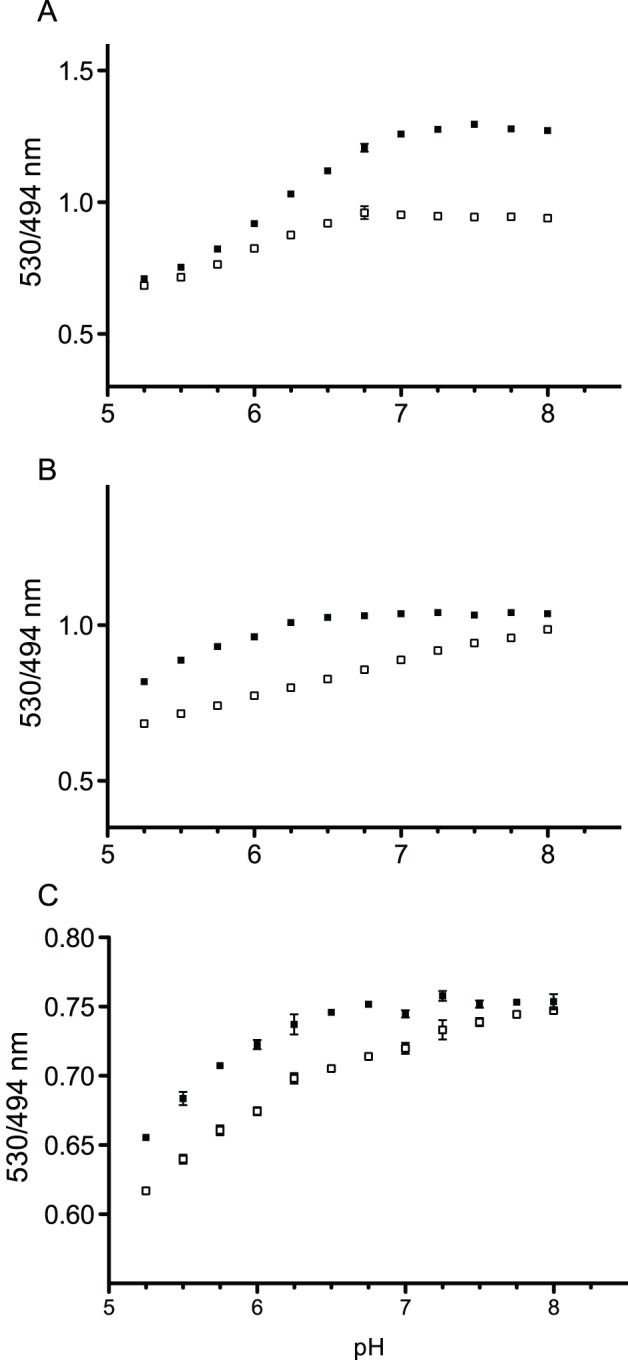
PH stability of FLIPQ-TV3.0_1.5 μ (A), 2 m (B) and 8 m (C) sensor. Venus/mTFP1 emission ratios were measured in MES-Tris buffer with altered pH, in the absence (black squares) or in the presence (open squares) of saturating concentrations of glutamine.

MTFP1, in addition to its better quantum efficiency compared to ECFP, is more stable at acidic pH (p*K*a 4.3) [31]. In order to examine the pH stability of FLIPQ-TV3.0 sensors, emission intensities in the pH range 5.25–8.0 were tested. FLIPQ-TV3.0_1.5 μ was found to be sensitive to acidic pH, with ΔR declining sharply below pH 6.5. However, above pH 6.75, both ΔR and emission intensities were fairly stable (Fig. 5). FLIPQ-TV3.0_2 m and 8 m, on the other hand, maintained a reasonable ΔR at more acidic pH, whereas ΔR declined at pH above 7.5. FLIPQ-TV sensors were more pH-stable compared to a similar chimeric protein that carries CFP in place of mTFP1 (Ahmad and Okumoto, unpublished results). Since the cytosolic pH of mammalian cells is considered to range between 7.0–7.4 [44], we concluded that the sensor is suitable for use in the cytosol of mammalian cells. The pH stability of these sensors makes them more suited for detecting transport processes that accompany cellular pH changes such as H^+^-symport, as in the case of system N-transporters, which has been proposed to mediate both the efflux and the uptake of glutamine [45].

### Live-cell Imaging of Cellular Glutamine Using FLIPQ-TV3.0 Sensors

In order to examine whether these FLIPQ-TV3.0 sensors function in live cells, the sensors were expressed in the cytosol of cos7 cells, a cell line that has been used for characterizing the activities of exogenous amino acid transporters in previous studies [46], [47]. In addition, the sensors were also expressed in SK-Hep cells, known to express a human neutral amino acid transporter ASCT2 which transports glutamine [48], [49]. FLIPQ-TV3.0 sensors were robustly expressed in the cytosol of both of the cell lines. FRET efficiency changes of FLIPQ-TV3.0_ 1.5 μ, 50 μ, 100 μ, 2 m and 8 m were tested by perfusing cells expressing an individual sensor with external glutamine, ranging from 50 µM to 5 mM. In cos7 cells, responses of all of these sensors to external glutamine applications were either undetectable or very weak and not reproducible (Fig. S4A-C). This could be due to low uptake capacity for glutamine relative to the activities of enzymes that maintain the glutamine concentration in the cells. On the other hand, a weak but reproducible response was observed in SK-Hep cells expressing FLIPQ-TV_8 m sensor (Fig.S5).

**Figure 6:**
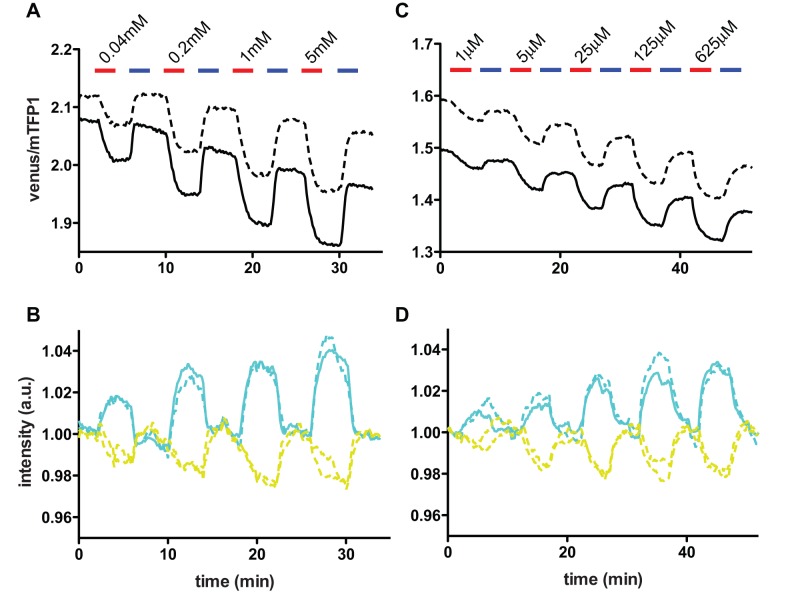
*In vivo* glutamine measurements using FLIPQ-TV3.0_8 m and 100 μ sensors. (A) The venus/mTFP1 ratio of cos7 cells co-expressing FLIPQ-TV3.0_8 m sensor and ASCT2-mCherry. The cells were perfused with HEPES-buffered Hank’s buffer. Timepoints when extracellular glutamine (red) and 5 mM Ala (blue) was added to the perfusion media are indicated as boxes above the graph. Solid and dashed lines represent two individual cells measured in the same experiment. (B) The intensities of mTFP1 and venus channels in the experiment shown in (A). The values were corrected for photobleaching and normalized to the baseline. (C) and (D) A similar experiment as in (A) and (B), performed with cos7 cells expressing the FLIPQ-TV3.0_100 μ sensor.

The weak endogenous activity of glutamine uptake in cos7 cells provides a suitable system to analyze the activity of exogenously expressed transporters. Therefore, we expressed ASCT2, tagged with a mCherry [50] in cos7 cells as a model of exogenous glutamine transporter. The ASCT2-mCherry construct localized mainly to the cytoplasm, consistent with previous reports [51], [52] (Fig. S6). When the FLIPQ-TV3.0_8 m was co-expressed with ASCT2-mCherry, a venus/mTFP1 ratio change was observed in the presence of extracellular glutamine, ranging between 40 µM and 5 mM (Fig. 6A and B), indicating that glutamine taken up by ASCT2 can be detected by the sensor expressed in the cytosol. The decrease in venus/mTFP1 ratio through the course of experiment is most likely due to the differential photobleaching of the two fluorophores [53]. The ratio change was concentration-dependent. A similar result was obtained using FLIPQ-TV3.0_2 m sensor (Fig. S7A). In contrast, cos7 cells co-expressing FLIPQ-TV3.0_1.5 μ, which is expected to be saturated in live cells due to its high affinity, did not respond to extracellular glutamine (Fig. S7B).

Interestingly, FLIPQ-TV3.0_100 μ also responded to the addition of glutamine ranging from 1 µM to 625 µM, saturating at lower concentrations (∼125 µM external glutamine) compared to the FLIPQ-TV3.0_2 m and 8 m sensors (Fig. 6C and D). This indicates that under such conditions, the cytosolic concentration of glutamine can fall down into the dynamic range of this sensor (∼100 µM if the concentration is down to the *Kd* of the sensor), which is much lower than the range of cellular glutamine concentrations reported so far (1–22 mM [27], [54], [55]). The ASCT2 transporter is reported to be overexpressed in cancerous cells [56], where it is assumed to have a role in meeting the increased demand for glutamine in cancerous cells. Indeed, it has been demonstrated that competitive inhibition of ASCT2-mediated glutamine uptake by Ala, Ser and Thr inhibits the growth of colon carcinoma [57] and hepatoma cell lines [58], [59], and accordingly, the possibility to target this transporter for pharmacological intervention has been suggested in the past [56], [60]. Since FLIPQ-TV3.0_100 μ can report the decrease in glutamine concentration into the sub-millimolar range, it would be possible to utilize the sensor in combination with chemical or RNAi libraries to identify a treatment that lowers cellular glutamine concentrations. In addition, duplex imaging of cellular glutamine concentration and the induction of apoptosis, using FLIPQ-TV sensors and a sensor for apoptosis, mAmetrine-DEVD-tdTomato [33] respectively, would offer an attractive method to correlate cellular glutamine concentration and the induction of apoptosis [61].

### Characterization of Glutamine Transporter Properties Using FLIPQ-TV Sensors

**Figure 7:**
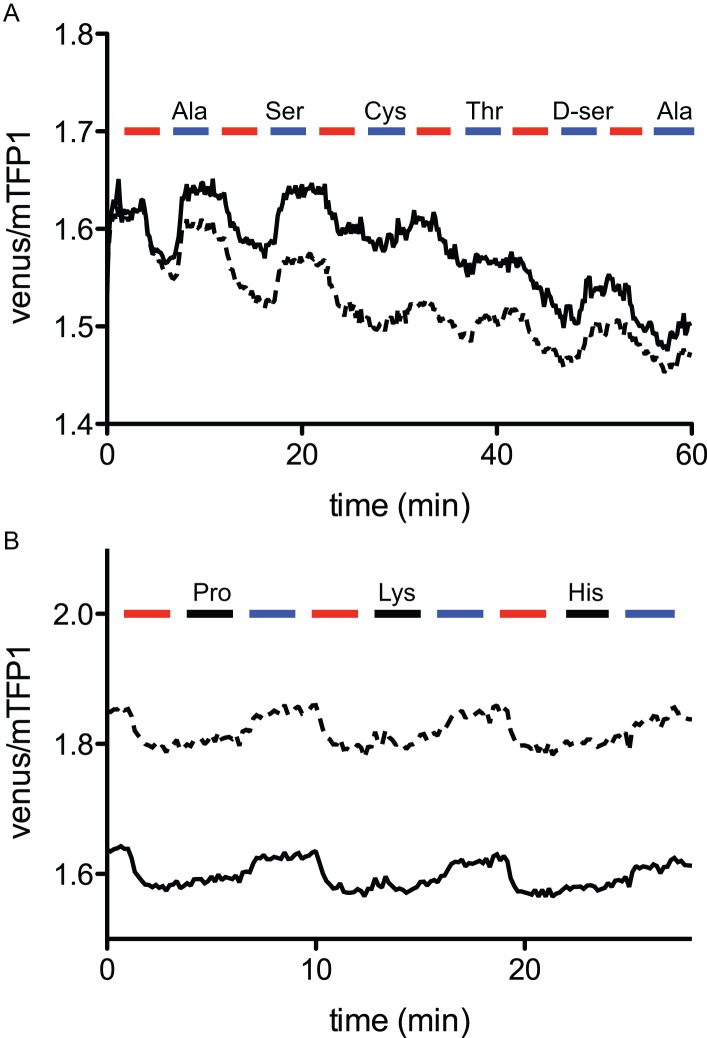
Elimination of cellular glutamine through the ASCT2 transporter in the presence of external amino acids, visualized using FLIPQ-TV3.0_8 m sensor. (A) Cytosolic glutamine is exported by the addition of extracellular Ala, Ser, Cys, Thr, and D-ser. Timepoints when extracellular glutamine (red boxes) or other amino acids (blue boxes) were added to the perfusion media are indicated as boxes above the graph. (B) Addition of Pro, Lys, His (filled boxes) does not alter cytosolic glutamine concentration, whereas the addition of Ala (blue boxes) promotes the export of glutamine. Solid and dashed lines represent two individual cells measured in the same experiment. All amino acids were added at 5 mM external concentrations.

**Figure 8:**
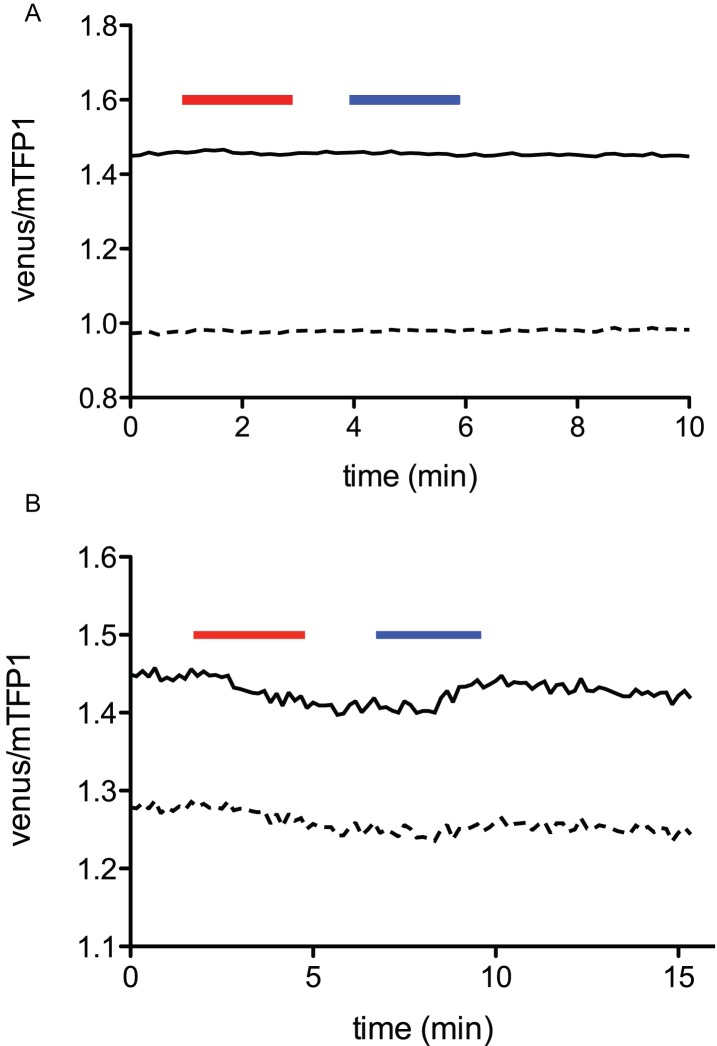
Responses of cos7 cells co-expressing the FLIPQ-TV3.0_8 m sensor and ASCT2-mCherry to external glutamine in the absence (A) or in the presence of 13.4 mM (∼10% of normal Hank’s buffer) of extracellular sodium. Timepoints when 5 mM extracellular glutamine (red box) or 5 mM Ala (blue box) are indicated as boxes above the graph. Solid and dashed lines represent two individual cells measured in the same experiment.

The cellular glutamine concentration stayed high after the removal of extracellular glutamine, indicating that the cellular metabolism is not sufficient to lower the level of glutamine to the detection range of these sensors. However, the addition of small, neutral amino acids such as Ala quickly reversed the cellular glutamine concentration (Fig. 6 and Fig. S7A). ASCT2 is an obligatory exchanger, and is able to mediate glutamine efflux in the presence of other extracellular amino acids [62]. Therefore we tested an array of amino acids for the ability to induce glutamine efflux. Ala, Thr, Cys, Ser, and D-ser, which are recognized by ASCT2, were able to induce glutamine efflux, whereas His, Pro, and Lys did not promote glutamine efflux (Fig. 7). The transport of glutamine was also dependent on extracellular sodium, consistent with previous observations [48] (Fig. 8). Since all of these results corroborate with the characteristics of ASCT2, we concluded that FLIPQ-TV sensors can be utilized to monitor cytosolic concentrations of glutamine, and that they can be used to investigate physiological parameters of the transporter, such as substrate specificity and dependence on extracellular sodium. Since genetically encoded sensors can be targeted to a single cell type using appropriate promoters, it would be possible to utilize these sensors to examine glutamine transport and metabolism in a specific cell type *in vivo.* Such an experimental setup will be particularly useful for tissues that consist of heterologous cell types with distinct glutamine metabolism (i.e. glial cells and neurons). For example, the molecular identity of the transporter that is responsible for providing glutamine to neuronal cells is still controversial in some cases [62], [63]. Presumably genetically encoded sensors could be utilized in combination with pharmacological techniques to analyze the transport system responsible for glutamine uptake in the context of live tissue, or even in an intact animal [64], [65], [66].

## Materials and Methods

### DNA Constructs

To construct FLIP-TV sensors, the coding sequence of *E.coli* glutamine binding protein (glnH, accession NC_000913) without the periplasmic leader sequence was amplified by PCR from genomic DNA of *E.coli* K12 with *Bam*HI and *Xho*I sites on the 5′- end and *Kpn*I and *Hind*III sites on the 3′-end respectively. Primers used were 5′- GAGGGATCCGCTCGAGGCAGGCTCGAAATTAGTTGTCGCGACGGATACCGCCTTCGTTCCGTTTGAATTTAAACAGGGCGCCAAATATGTGGGCTTTGAC -3′ (underlined sequence indicates the *Sfo*I site introduced for the insertion of mTFP1) and 5′- GAGAAGCTTGGGTACCTTTCGGTTCAGTACCGAACC -3′. The resulting PCR product was cloned into the *Bam*HI/*Hind*III site of pRSET B vector (Invitrogen), producing pRSET-glnH. The mTFP1 sequence with *Eco47*III sites on both ends was amplified from pBAD/HisB-mTFP1 (generous gift from Dr. Campbell) using primers 5′– GAGAGCGCTGGTATGGTGAGCAAGGGCGAGGAGCTG –3′ and 5′- GAGAGCGCTACCGTACAGCTCGTCCATGCCGAGAGT -3′. The resulting PCR product was digested with *Eco47*III and cloned into the *Sfo*I site of pRSET-glnH. The venus sequence with *Kpn*I at the 5′-end and *Hind*III at the 3′- was digested from pRSET-FLIPEWT [40] and fused in-frame to the 3′- end of the glnH sequence. The resulting construct was named pRSET-FLIPQTV. Point mutants were created by site-directed mutagenesis [67]. For the optimization of linker sequences between the fluorophores and binding protein, the following primers were used: 5′- GCCCTTGCTCACCATNNNNNNCTGTTTAAATTCAAAC -3′ (Fig. 3A, Ln1), 5′- GTCAAAGCCCACATATTTGGCGCTNNNCTTGTACAGCTCGTC -3′ (Fig. 3A, Ln2), and 5′- CCCGGTGAACAGCTCNNNNNNTTTCGGTTCAGTACC -3′ (Fig. 3A, Ln3).

A plasmid containing human *ASCT2* (ATCC MGC 1387) was purchased from the American Type Culture Collection (ATCC). The *ASCT2-monomeric Cherry (mCherry)* fusion was made by PCR-stitching. The *ASCT2* sequence without the stop codon, followed by a linker sequence was amplified using a forward primer *ASCT2*-*F*: 5′-GAGAAGATCTCGGTGCTTCCCATCATGGTGGCCGATCCTCCTCGAG -3′ and reverse primer *ASCT2*-*R*: 5′- GGCGGGATCTCCTCCACCGCCCCCTCCCATGACTGATTCCTTCTCAGAGGC -3′. The *mCherry* sequence was amplified using a forward primer *mCherry*-*F*: 5′- GGGGGCGGTGGAGGAGATCCCGCCACCATGGTGAGCAAGGGCGAGGAGGAT -3′ and a reverse primer *mCherry*-*R*: 5′- GAGAGAATTCCTTACTTGTACAGCTCGTCCATGCCGCC -3′. The underlined sequences represent a complementary region between two fragments. The two PCR fragments were extended in the second PCR cycle with *ASCT2-F* and *mCherry-R* primers. The resulting *ASCT2-mCherry* fusion fragment was digested with *Bgl*II and *Eco*RI, then cloned in between *Bam*HI and *Eco*RI sites in pENTR1A vector (Invitrogen). The *ASCT2-mCherry* sequence was recombined into pcDNA3.2 V5-DEST vector (Invitrogen) using LR-clonase II, following the manufacturer’s instruction.

### Cell Cultures

Cos7 (ATCC CRL-1561) and SK-Hep (ATCC HTB-52) cells were purchased from ATCC. Cells were cultured in Dulbeccos Modified Eagles Medium supplemented with 5% cosmic calf serum, 100 U/ml penicillin, and 100 U/ml streptomycin. Cells were cultured at 37°C under an atmosphere of 5% CO_2_. For imaging, cells were plated on an 8-well chamber with glass bottom, coated with poly-L-lysine. Transfection was performed using Lipofectamine 2000 (Invitrogen). For transfecting cells, 400 ng of *FLIPQ-TV* constructs and 1.2 µg of *ASCT2-mCherry* construct/well (100 mm^2^) were used.

### High-throughput Protein Purification

BL21 (DE3) gold (Stratagene) colonies expressing randomly mutagenized FLIPQ-TV sensors were cultured in 2 ml of SB medium in 96 well plates. Cells were lysed with a sonicator equipped with a 96-pin tip, and purified using 96 well Ni-NTA columns (Qiagen). Emission spectra for each sample in the presence and absence of glutamine were measured with excitation at 450 nm using a plate reader (Synergy4, BioTek), and clones with larger changes in mTFP1 and venus peaks (494 and 535 nm respectively) were kept for further analysis.

### In vitro Characterization of FLIPQ Sensors

Bacterial expression constructs for FLIPQ sensors were introduced into *E.coli* BL21(DE3) Gold (Stratagene), and the expressed sensor proteins were purified using Ni-NTA columns as described in [68]. Ligand titration curves were obtained by using a microplate reader (Synergy 4, BioTek), with excitation at 450 nm and emission at 490 nm and 535 nm for mTFP1 and venus, respectively. To determine the *Kd* of each FLIPQTV sensor, sensor protein was mixed with different concentrations of glutamine in 20 mM HEPES buffer, pH 7.0, and venus/mTFP1 ratio was measured. The *Kd* was determined by fitting the saturation curve to a single site binding isoterm: *S = *(*r-r_apo_*)*/*(*r_sat_ – r_apo_*)* = *[*L*]/([*Kd*]+[*L*]), where *S* is saturation, [*L*] is ligand concentration, *r* is ratio, *r_apo_* is the ratio without ligand, *r_sat_* is the ratio at saturation. Saturation curves were obtained from at least three independent protein preparations.

### In vivo Imaging of Cell Cultures

Cells were imaged 36–72 hrs post-transfection using an epifluorescence microscope (IX81, Olympus) with appropriate filters (mTFP1 excitation: 455/10, mTFP1 emission: 495/30, venus excitation: 470/24, venus emission: 535/30, mCherry excitation: 560/40, mCherry emission: 630/75). The cells were perfused with Hank’s balanced saline solution (HBSS), supplemented with 25 mM HEPES and 4.2 mM sodium bicarbonate, pH 7.35. Prior to the time-course experiments, the cells were perfused with 5 mM Ala for 2 min to lower the concentration of intracellular glutamine, then washed briefly with HBSS. The images were collected and analyzed using appropriate software (Slidebook, 3I). For testing transporter activities in Na^+^ - free buffer, sodium components in HBSS were replaced with choline, and the cells were incubated for 15 min in this choline-based buffer prior to perfusion with amino acids.

## Supporting Information

Figure S1 The surface views of glnH in open- and closed- forms. (A) Alignment between the open- (blue, 1GGG) and closed- (beige, 1WDN) structures, shown in ribbon diagrams. Glutamine molecule in the cleft is represented as ball-and-stick. C-termini, where the venus protein is fused in FLIPQ-TV1.0 is marked in magenta. (B) and (C) The surface views of the open- (B) and closed (C) structures, superimposed on the ribbon diagram shown in (A). Note the large change in spatial constraint in the vicinity of C-termini (represented in magenta).
(TIF)

Figure S2 Optimization of FLIPQ-TV sensors. The linkers (Ln1–3) were iteratively mutagenized, and clones representing individual mutagenesis events were screened for an improved ΔR/R_0_. The number of clones screened on each step, and ΔR/R_0_ of each generation of sensors are indicated.
(EPS)

Figure S3 Residues that were altered to produce affinity mutants. (A) Ball-and-stick representation of the glutamine molecule in the cleft and residues in the cleft likely to form hydrogen bonds with the glutamine. (B) The location of Tyr86 and Trp220 that are mutated in FLIPQ-TV3.0_2 m and 8 m.
(EPS)

Figure S4 Cos7 cells expressing FLIPQ-TV3.0_100 μ (A), 2 m (B), 8 m (c) sensors perfused with extracellular glutamine. The cells were perfused with HEPES-buffered Hank’s buffer. Timepoints when extracellular glutamine (red) and 5 mM Ala (blue) were added to the perfusion media are indicated as boxes above the graph. Solid and dashed lines represent two individual cells measured in the same experiment.
(EPS)

Figure S5 A SK-Hep cell expressing FLIPQ-TV3.0_8 m sensor perfused with extracellular glutamine. Timepoints when 5 mM glutamine (red) or 5 mM Ala (blue) were added to the perfusion media are indicated as boxes above the graph.
(EPS)

Figure S6 Cos7 cells co-expressing ASCT2-mCherry fusion (red) and FLIPQ-TV3.0_8 m (green). ASCT2 signal is mostly found on the endomembrane, whereas FLIPQ-TV3.0_8 m is distributed throughout the cytosol.
(EPS)

Figure S7 *In vivo* glutamine measurement using FLIPQ-TV3.0_2 m sensor. (A) Venus/mTFP1 ratio of cos7 cells co-expressing FLIPQ-TV3.0_2 m sensor and hASCT2-mCherry. The cells were perfused with HEPES-buffered Hank’s buffer. Timepoints when extracellular glutamine or alanine (5 mM) were added to the perfusion media are indicated as red and blue boxes above the graph. Solid and dashed lines represent two individual cells measured in the same experiment. (B) The intensities of mTFP1 and venus channels in the experiment shown in (A). (C) and (D) Venus/mTFP1 ratio and intensities of mTFP1 and venus channels of cos7 cells co-expressing FLIPQ-TV3.0_1.5 μ sensor and hASCT2-mCherry. Timepoints when extracellular glutamine and alanine (5 mM) were added are indicated as in (A).
(TIF)

Table S1 A complete list of mutations in glnH that were tested for altered affinity.
(DOCX)
