# Correction: Visualization of Glutamine Transporter Activities in Living Cells Using Genetically Encoded Glutamine Sensors

**DOI:** 10.1371/journal.pone.0140088

**Published:** 2015-10-05

**Authors:** Katrin Gruenwald, John Todd Holland, Verlyn Stromberg, Altaf Ahmad, Daisy Watcharakichkorn, Sakiko Okumoto

The seventh sentence of the first paragraph under the subheading “Affinity and Substrate Specificities of FLIPQ-TV3.0 Sensors” of the Results and Discussion section is incorrect. The correct sentence is: The resulting clones, FLIPQ-TV3.0_R75K, R75M, D157N, R75MW220A, and R75MY86A had *Kd* of 1.5×10−6 M, 5.3×10−5 M, 1.3×10−4 M, and 1.6×10−3, and 7.6×10−3 M, respectively (Fig 4A, Table 1).

**Table 1:** Affinities of FLIPQ-TV3.0 point mutants.

Mutations in glnH	*Kd*	R_apo_	R_sat_	ΔR/R_0_
WT	85nM	1.09	1.05	0.03
R75K	1.5μM	1.23	0.90	0.26
R75M	50μM	1.05	0.95	0.10
D157N	130μM	1.34	0.99	0.26
R75MW220A	1.6mM	1.29	1.13	0.12
R75MY86A	7.6mM	1.14	1.01	0.11

**Fig 4:**
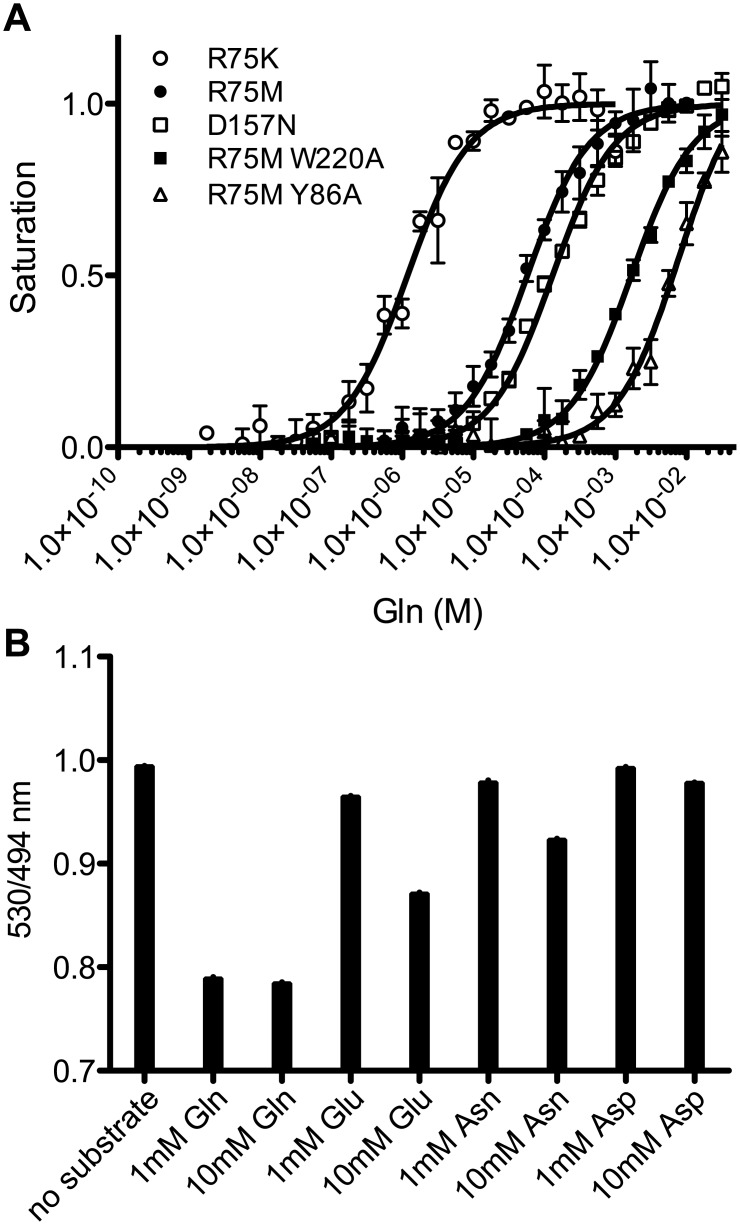
Affinities and substrate specificities of FLIPQ-TV3.0 sensors. (A) Saturation curves of FLIPQ-TV3.0 sensors with altered affinities. (B) Substrate specificities of FLIPQ-TV3.0_1.5 μ (black), 50 μ (hatched), 100 μ (white), 2 m (horizontal stripes), and 8 m (gray) sensors to Gln, Glu, Asn and Asp.

In Table 1, the fifth and six row labels under the column heading Mutations in glnH are swapped. Please see the corrected Table 1 here.

In Fig 4A, the in-figure legends for R75MW220A and R75AY86A are swapped. Please view the correct Fig 4 here.
