# Supplementary figures and images for: Visualization of Glutamine Transporter Activities in Living Cells Using Genetically Encoded Glutamine Sensors
Source: PLoS One. 2012 Jun 14;7(6):e38591. doi: 10.1371/journal.pone.0038591 (PMC3375291; doi:10.1371/journal.pone.0038591)

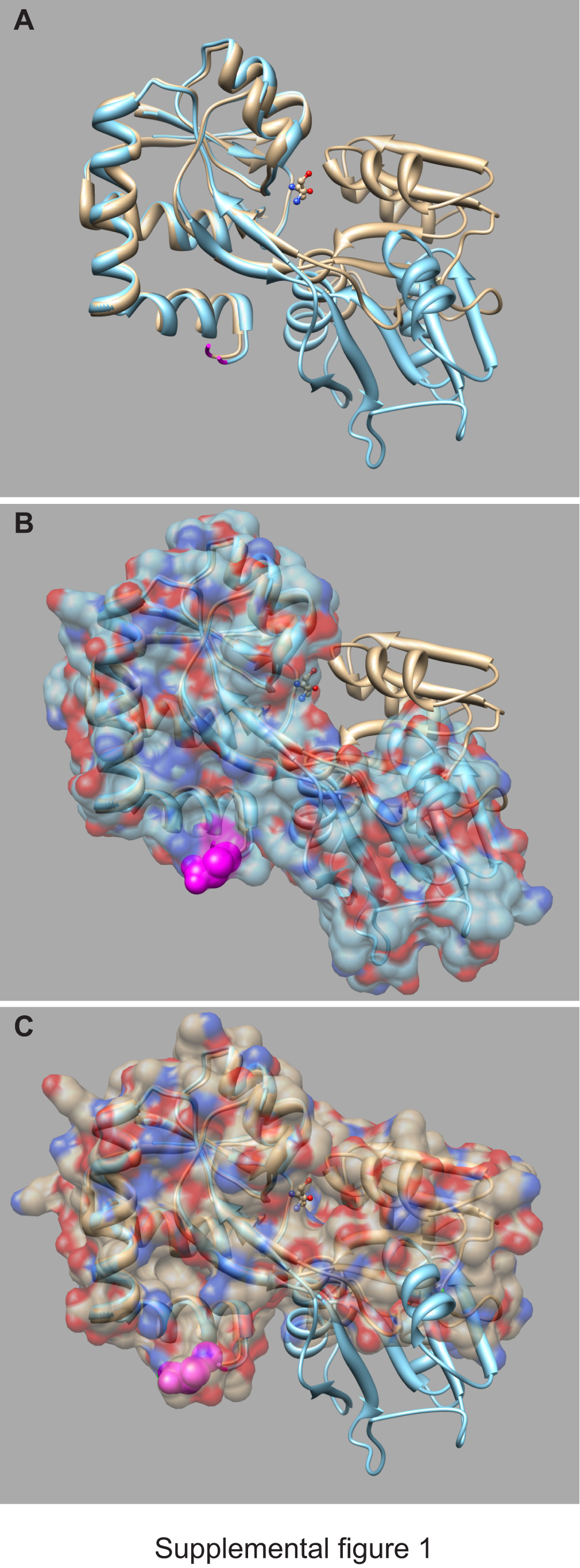

Supplement: Figure S1 — The surface views of glnH in open- and closed- forms. (A) Alignment between the open- (blue, 1GGG) and closed- (beige, 1WDN) structures, shown in ribbon diagrams. Glutamine molecule in the cleft is represented as ball-and-stick. C-termini, where the venus protein is fused in FLIPQ-TV1.0 is marked in magenta. (B) and (C) The surface views of the open- (B) and closed (C) structures, superimposed on the ribbon diagram shown in (A). Note the large change in spatial constraint in the vicinity of C-termini (represented in magenta). (TIF) [file pone.0038591.s001.tif]

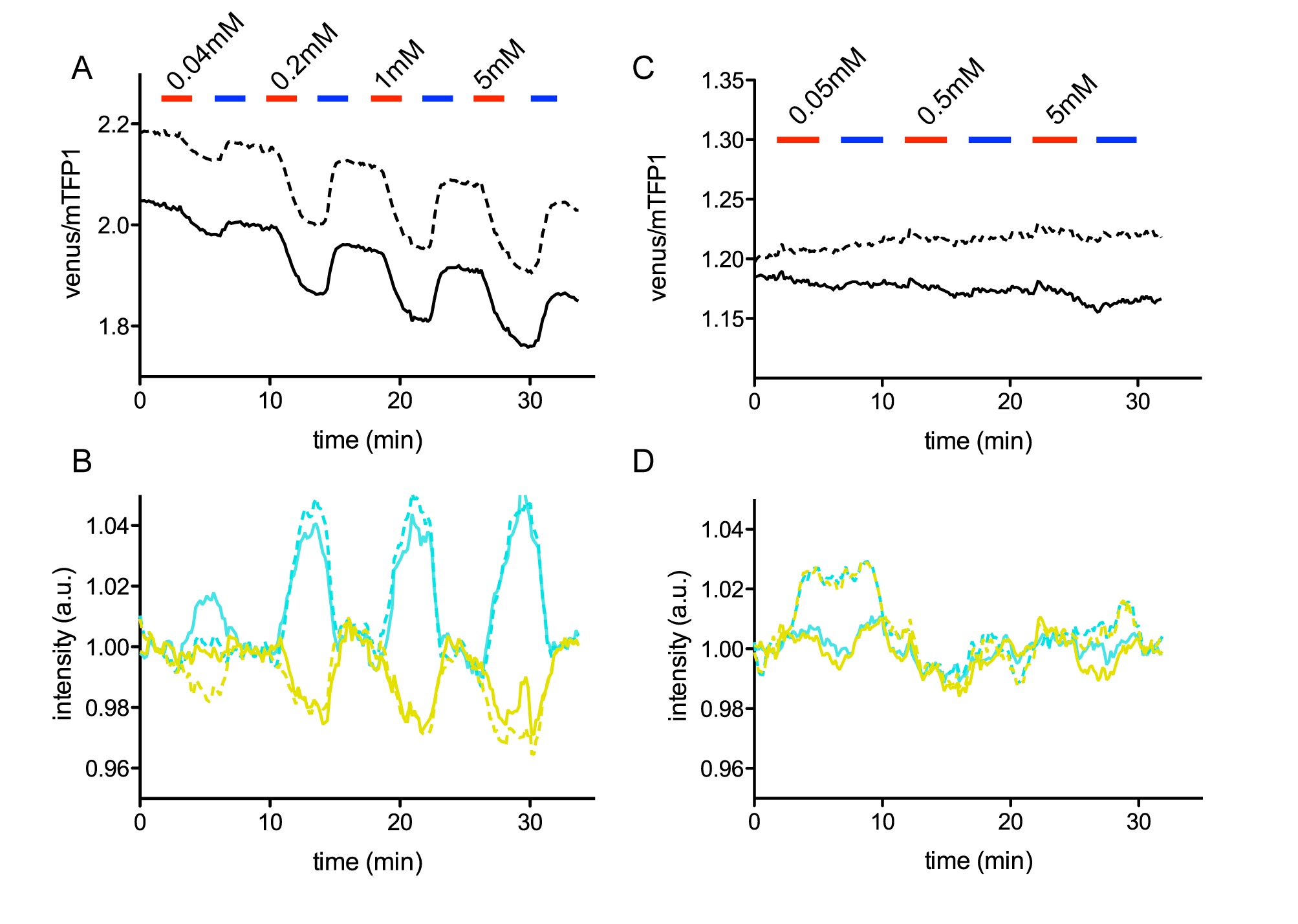

Supplement: Figure S7 — In vivo glutamine measurement using FLIPQ-TV3.0_2 m sensor. (A) Venus/mTFP1 ratio of cos7 cells co-expressing FLIPQ-TV3.0_2 m sensor and hASCT2-mCherry. The cells were perfused with HEPES-buffered Hank’s buffer. Timepoints when extracellular glutamine or alanine (5 mM) were added to the perfusion media are indicated as red and blue boxes above the graph. Solid and dashed lines represent two individual cells measured in the same experiment. (B) The intensities of mTFP1 and venus channels in the experiment shown in (A). (C) and (D) Venus/mTFP1 ratio and intensities of mTFP1 and venus channels of cos7 cells co-expressing FLIPQ-TV3.0_1.5 μ sensor and hASCT2-mCherry. Timepoints when extracellular glutamine and alanine (5 mM) were added are indicated as in (A). (TIF) [file pone.0038591.s007.tif]
