# Supplementary material for: Visualization of Glutamine Transporter Activities in Living Cells Using Genetically Encoded Glutamine Sensors
Source: PLoS One. 2012 Jun 14;7(6):e38591. doi: 10.1371/journal.pone.0038591 (PMC3375291; doi:10.1371/journal.pone.0038591)
Supplement: Table S1 — A complete list of mutations in glnH that were tested for altered affinity. (DOCX) [file pone.0038591.s008.docx]

| Point mutations | *Kd* | R_apo_ | R_sat_ | ΔR/R_0_ |
| --- | --- | --- | --- | --- |
| WT | 85nM | 1.09 | 1.05 | 0.03 |
| R75K | 1.5μM | 1.23 | 0.90 | 0.26 |
| R75KF13A | no apparent binding |  |  |  |
| R75KY86A | 300μM | 1.09 | 1.02 | 0.07 |
| R75KK219A | 400nM | 0.93 | 0.87 | 0.06 |
| R75KW220A | 20μM | 0.91 | 0.87 | 0.04 |
| R75M | 50μM | 1.05 | 0.95 | 0.10 |
| R75MY86A | 1.6mM | 1.29 | 1.13 | 0.12 |
| R75MW220A | 7.6mM | 1.14 | 1.01 | 0.11 |
| D157N | 100μM | 1.34 | 0.99 | 0.26 |
